# Restoration of Mismatch Repair Functions in Human Cell Line Nalm-6, Which Has High Efficiency for Gene Targeting

**DOI:** 10.1371/journal.pone.0061189

**Published:** 2013-04-15

**Authors:** Tetsuya Suzuki, Akiko Ukai, Masamitsu Honma, Noritaka Adachi, Takehiko Nohmi

**Affiliations:** 1 Division of Genetics and Mutagenesis, National Institute of Health Sciences, Setagaya-ku, Tokyo, Japan; 2 Biological Safety Research Center, National Institute of Health Sciences, Setagaya-ku, Tokyo, Japan; 3 Graduate School of Nanobioscience, Yokohama City University, Yokohama, Kanagawa, Japan; Louisiana State University and A & M College, United States of America

## Abstract

Gene targeting is a powerful approach in reverse genetics. The approach has been hampered in most of human cell lines, however, by the poor targeting efficiency. Nalm-6, a human pre-B acute lymphoblastic leukemia cell line, exhibits exceptionally high gene targeting efficiency and is used in DNA repair and the related research fields. Nonetheless, usage of the cell line is still limited partly because it lacks expression of MSH2, a component of mismatch repair complex, which leads to increased genome instability. Here, we report successful restoration of MSH2 expression in Nalm-6 cells and demonstrate that the recovery does not affect the high targeting efficiency. We recovered the expression by introduction of cDNA sequences corresponding to exons 9 to 16 at downstream of exon 8 of the *MSH2* gene. Endogenous exons 9 to 16 were deleted in the cell line. The MSH2 expression substantially reduced spontaneous *HPRT* mutation frequency. Moreover, gene targeting efficiency in the MSH2-expressing cells was similar to that in the MSH2-lacking cells. In fact, we generated heterozygously *REV3L* knockout and the catalytically dead mutants in the MSH2-proficient Nalm-6 cells with efficiency of 20–30%. The established cell line, Nalm-6-MSH+, is useful for reverse genetics in human cells.

## Introduction

Gene targeting technology, which introduces novel DNA sequences at specific sites of the chromosome with exogenous DNA, is a powerful tool to investigate gene functions. In human cell lines, however, the method is not practicable in most cases because efficiency of homologous recombination (HR) necessary for the gene targeting is much lower than that of non-homologous end joining (NHEJ) [1], [2]. The inefficiency makes targeted recombinants mostly hidden by predominant non-targeted random integrants. Thus, knockdown of gene expression by small interfering (si) RNA has been utilized widely to examine cellular functions of genes of interest in human cells. However, reduction of gene expression by siRNA is not perfect and 10–20% of gene expression usually remains after siRNA treatments. Recent development of zinc-finger nucleases (ZFNs) [3], which introduce double-strand breaks at unique chromosome sites and enhance local mutagenic-NHEJ and HR, appears a good candidate to overcome the limitation [4], [5]. Nevertheless, design and selection of the zinc-finger domains that recognize unique sites of the chromosome are not easily predictable *in silico.* Hence, establishment of ZFNs often requires *in vivo* experiments to ensure that the designed enzymes do not introduce double-strand breaks at off-target sites. Ready-to-use ZFNs are commercially available, but numbers of genes that can be manipulated by the commercial ZFNs are still limited and made-to-order commercial ZFNs are costly.

Interestingly, several human cell lines possess exceptionally high gene targeting efficiency. Nalm-6, a pre-B acute lymphoblastic leukemia cell line, is one of them and the ratio between homologous recombinants and random integrants, i.e., targeting efficiency, is reported 1–30% [6]–[10]. The efficiency might be comparable to that of chicken B-lymphocyte DT40, which is frequently used to examine functions of various genes involved in DNA repair, recombination and translesion DNA synthesis (TLS) [11]. In addition, Nalm-6 has a stable near-diploid karyotype with normal p53 status. Genes involved in double-strand break repair have been knocked out in this cell line [6].

However, Nalm-6 lacks the expression of MSH2, a component of mismatch repair proteins [6], [12]. MSH2 forms two distinct heterodimers, i.e., MutSα (MSH2/MSH6) and MutSβ (MSH2/MSH3) in human cells, both of which play a critical role in recognition of mismatch bases in DNA and initiation of repair [13]. MutSα recognizes base-base mismatches and deletion/insertion of one or two bases, while MutSβ preferentially recognizes larger insertion/deletion mispairs [14]. Defect of MSH2 leads to genome instability and extremely high spontaneous mutation frequency [15]. In addition to mismatch bases, both MutS complexes are able to bind to a wide variety of lesions in DNA, e.g., alkylated bases in DNA, oxidative DNA damage [16], UV photoproducts [17] and DNA-crosslinks [18], suggesting that the complexes may act as a general sensor of DNA damage, which initiates downstream cellular responses such as apoptosis [19]. Therefore, cells deficient in MSH2 may exhibit abnormal responses against DNA damaging agents in comparison to cells with the repair functions.

In this study, we analyzed the cause of deficiency of MSH2 expression and restored the expression of MSH2 in Nalm-6. The restoration led to stable expression of MSH6, which makes a complex with MSH2. In Nalm-6 cells, the MSH6 gene is intact but the protein is poorly expressed because of the lack of MSH2 expression [6], [20]. The Nalm-6 cells expressing MSH2/MSH6, which we call Nalm-6-MSH+ hereafter, displayed substantially lower spontaneous mutation frequency and higher cytotoxicity against an alkylating agent, *N*-methyl -*N*′-nitro-*N*-nitrosoguanidine (MNNG) than the original Nalm-6 in the presence of an inhibitor of *O* ^6^-methylguanine-methyltransferase, i.e., *O* ^6^-benzylguanine (*O* ^6^-BG). Furthermore, we revealed that the expression of MSH2 had no effects on gene targeting efficiency. It suggests that the lack of mismatch repair functions is not a cause of high gene targeting of this cell line and also that efficient manipulation of genome is possible in the cells expressing MSH2/MSH6. Nalm-6-MSH+ is useful for detailed analyses of functions of genes in responses to DNA damaging agents in human cells.

## Materials and Methods

DNA sequences of all primers used for amplification by polymerase chain reactions (PCR) and site-directed mutagenesis are described in Table S1.

### Cell Culture and Transfection

Nalm-6 [6]–[10] was cultured in RPMI1640 supplemented with 10% calf serum and 50 µM 2-mercaptoethanol at 37°C under a 5% CO_2_ atmosphere. Cells were transfected with DNA constructs using Nucleofector I (LONZA) according to the manufacturer’s protocol. Briefly, 2×10^6^ cells were suspended into 0.1 mL of KitT solution with supplement 1, and transfected with 2 µg of a linearized targeting vector by using Program C-05. Transfected cells were cultured for 48 h at 37°C and then the optimum numbers of the cells were seeded into 96-well plates in medium containing an appropriate drug (G418; 600 µg/mL, Puromycin; 0.5 µg/mL, Hygromycin B; 400 µg/mL). The drugs were purchased from Sigma-Aldrich, Wako and Life Technologies, respectively. Gene targeting vectors were constructed by using MultiSite Gateway Three-Fragment Vector Construction Kit (Life Technologies) as previously described [21].

### Comparative Genome Hybridization (CGH) Array Analyses

Genomic DNA was extracted with Gentra Puregene Core Kit A (QIAGEN). The genome of human lymphoblastic cell line TK6 [22], [23] was used as control. Genomic DNA was digested with *Alu*I and *Rsa*I, and then labeled with Genomic DNA Labeling Kit Plus (Agilent Technologies). Labeled DNA was purified with Microcon YM-30 (Millipore Corporation). The sample genomes were hybridized with Human Genome CGH 244 K Microarray slide (Agilent Technologies). The array slide was scanned by GenePix 4000 B (Axon Instruments Inc.) and analyzed with DNA Analytics ver 4.0 (Agilent Technologies).

### Establishment of Nalm-6-MSH+

Two loxP sites in pENTR lox-Hyg were replaced with different mutant loxP (mloxP) sequences (lox66 and lox71) [24], [25] (Method S1). The hygromycin-resistance gene was replaced with the neomycin-resistance gene. The resulting plasmid was named pENTR mloxP-Neo. All PCR reactions were performed with KOD FX (TOYOBO). *MSH2* genomic fragment containing the region between a splicing acceptor of intron 8 and exon 9 was amplified by PCR using TK6 genomic DNA as template and primers of MSH2-I8 BamHI-Fw and MSH2-E9 Rv. The DNA fragment was digested with *Eco*RI/*Bam*HI and the digested DNA was ligated into pBluescript II SK(+) (Agilent Technologies) at the same sites. The resulting plasmid was named pBluescript II SK(+)-MSH2 I8-E9. *MSH2* cDNA fragments containing the region between exon 9 and exon 16 were amplified by reverse transcription (RT)-PCR with TaKaRa RNA PCR Kit (AMV) Ver.3.0 using TK6 total RNA as template and primers of MSH2-E8 Fw and MSH2-E16 XhoI-Rv, and ligated into pBluescript II SK(+)-MSH2 I8-E9 at the *Eco*RI/*Xho*I sites. The resulting plasmid was named pBluescript II SK(+)-MSH2 I8-E16. *MSH2* genomic fragments containing the region between exon 16 and 3′-untranslated region (3′-UTR) were amplified by PCR using TK6 genomic DNA as template and primers of MSH2-E16 Fw and MSH2-3′UTR XhoI-Rv, and ligated into pBluescript II SK(+)-MSH2 I8-E16 at the *Hpa*I/*Xho*I sites. The plasmid DNA was digested with *Bam*HI/*Xho*I. The resulting DNA fragment containing the region from the splicing acceptor site of intron8 to the 3′-UTR was blunt-ended and subcloned into pENTR mloxP-Neo at the blunt-*Eco*RI site. Genomic fragments surrounding intron 8 of *MSH2* with the DNA size of 2.4- and 3.0-kb, respectively, were amplified by PCR using Nalm-6 genomic DNA as template and were used as 5′- and 3′- arms, respectively, of MultiSite Gateway system. Two primer sets of MSH2-5′arm Fw and Rv and MSH2-3′arm Fw and Rv were used to amplify 5′-arm and 3′-arm, respectively. pENTR mloxP-Neo containing the splicing acceptor, cDNA of exon 9 to exon 16 and the 3′-UTR of the *MSH2* gene, two plasmid DNAs containing 5′-arm or 3′-arm and pDEST DTA-MLS were mixed to generate a targeting vector to restore MSH2 expression in Nalm-6 cells according to the protocol of MultiSite Gateway system. Then, the targeting vector was linearized with *Pme*I and the linearized DNA was transfected into Nalm-6 cells. The transfected cells were selected in the medium containing G418 as described above. Targeted clones were identified by PCR screening using primers of MSH2 GT-Fw and 3′-loxP. *MSH2* mRNA was confirmed by RT-PCR with TaKaRa RNA PCR Kit (AMV) Ver.3.0 using primers of MSH2-E8 Fw and MSH2-E16 Rv. The neomycin-resistance gene was excised by introduction of Cre recombinase expression vector by Nucleofector I.

### Western Blotting

The cells were extracted with PRO-PREP (iNtRON) according to the supplier’s recommendations. The whole cell extracts were fractionated on 10% sodium dodecyl sulfate polyacrylamide gel and transferred to polyvinylidene difluoride membranes. The membranes were blocked with 5% non-fat milk and probed with either a mouse anti-MSH2 monoclonal antibody (SANTA CRUZ BIOTECHNOLOGY) or a mouse anti-MSH6 monoclonal antibody (SANTA CRUZ BIOTECHNOLOGY) overnight in 5% non-fat milk solution. After washing three times with phosphate-buffered saline containing 0.05% Tween 20, the membranes were incubated with an anti-mouse IgG conjugated to horseradish peroxidase (GE Healthcare Bio-Sciences) for 1 h. The MSH2 and MSH6 were then visualized using the Enhanced Chemiluminescence (ECL) System (GE Healthcare Bio-Sciences).

### *HPRT* Mutation Assays

Cells were treated with CHAT medium, which contained 10 µM deoxycytidine, 200 µM hypoxanthine, 0.1 µM aminopterin, and 17.5 µM thymidine, to reduce the background mutant fraction and then cultured with normal medium for 7 days to permit generation of spontaneous *HPRT*-deficient mutants. To isolate the mutants, cells were seeded into 96-well plates at 8000 or 40000 cells/well in the presence of 0.5 µg/mL 6-thioguanine. The plates were incubated for 18∼21 days at 37°C and then scored for colonies. Mutant frequencies were calculated based on the Poisson distribution [26].

### Cytotoxicity Assay with MNNG

We prepared 100 µl aliquots of cell suspension at a concentration of 2.0×10^5^ cells/mL in 96-well plates in the absence or the presence of 5 µM *O* ^6^-BG (Sigma-Aldrich). MNNG (Wako) dissolved in dimethyl sulfoxide was added to the wells at various concentrations and the plates were incubated for 48 h at 37°C. At the end of the treatment period, 10 µL of Cell Counting Kit-8 (DOJINDO) was added into each well and the plates were incubated for 4 h at 37°C. After the incubation, the absorbance at 450 nm was measured and the cell viability was calculated. The absorbance is proportional to the number of living cells.

### Gene Targeting Efficiency

To construct a targeting vector for knockout of the *HPRT* gene, genomic fragments of 5′- and 3′-side of exon 7 of the *HPRT* gene with the size of 2.4- and 2.8-kb, respectively, were amplified by PCR from Nalm-6 genomic DNA and used as the 5′- and 3′-arms of MultiSite Gateway system. Two primer sets of HPRT KO-5′arm Fw and Rv and HPRT KO-3′arm Fw and Rv, were used to amplify 5′-arm and 3′-arm, respectively. To examine how mismatch repair functions affect microheterogeneity in the homology arms, four mutations creating restriction sites (*Bam*HI, *Eco*RI, *Xho*I, *Hin*dIII) were introduced into the 5′-arm of the *HPRT* targeting vector by QuikChange Lightning Multi Site-Directed Mutagenesis Kit (Agilent Technologies) using four mutagenic primers, i.e., HPRT 5′arm *Bam*HI, *Eco*RI, *Xho*I and *Hin*dIII. These DNA fragments were combined with pENTR loxP-Puro and pDEST DTA-MLS by MultiSite Gateway system to generate the targeting vector for the *HPRT* gene, which was then linearized by *Pme*I and transfected into Nalm-6 as described above. As control, another targeting vector without restriction sites was also generated. Transfected cells were cultured for 48 h at 37°C and then the optimum numbers of the cells were plated onto 96-well plates in medium containing 0.5 µg/mL puromycine. After 7 days of culture, 6-thioguanine (final concentration; 0.5 µg/mL) was added for selection of *HPRT* mutants. Correctly targeted clones were confirmed by PCR using primers HPRT-KO GT-Fw and 5′-loxP, and the products were digested with restriction enzymes to confirm introduction of the mutations in the 5′-arm. Gene targeting frequency were calculated based on the ratio of a plating efficiency of puromycin- and 6-thioguanine-double resistant cells (correctly targeted clones) and that of puromycin-single resistant cells (randomly integrated clones).

### Establishment of *REV3L* Knockout and Knock-in Cells using Nalm-6-MSH+

To construct a targeting vector for knockout of the *REV3L* gene in one allele, 2.1- and 2.2-kb DNA regions located 5′- and 3′-side of exon 5 of *REV3L* were amplified by PCR using Nalm-6 genomic DNA as template and were used as 5′- and 3′-arms for the targeting vectors. Two primer sets of REV3 KO-5′arm Fw and Rv and REV3 KO-3′arm Fw and Rv, were used to amplify 5′-arm and 3′-arm, respectively. These DNA fragments were combined with pENTR loxP-Hyg and pDEST DTA-MLS by MultiSite Gateway system. To construct targeting vectors for knock-in of a catalytically-dead mutation in exon 30 of the *REV3L* gene, 2.0-kb DNA regions surrounding exon 30 were amplified by PCR and were used as 5′- and 3′-arms for the targeting vectors. Two primer sets of REV3 CD-5′arm Fw and Rv and REV3 CD-3′arm Fw and Rv were used to amplify 5′-arm and 3′-arm, respectively. The catalytically dead mutation, i.e., GGCGATACTGACAG to GGCGCCACTGCCAG, which directs amino acid changes of aspartate 2781 to alanine and aspartate 2783 to alanine (D2781A/D2783A), was introduced into the 3′-arm by QuikChange Lightning Site-Directed Mutagenesis Kit (Agilent Technologies) using mutagenic primers D2781/3A NarI-S and D2781/3A NarI-AS. Introduction of the catalytically dead mutation created a new restriction site of *Nar*I. The arms were combined with pENTR loxP-Hyg and pDEST DTA-MLS. The resulting targeting vectors containing the hygromycin-resistance gene was linearized by *Pme*I and transfected into Nalm-6 as described above. The transfected cells were cultured for 48 h at 37°C and then the optimum numbers of the cells were plated onto 96-well plates in medium containing 400 µg/mL hygromycin. After 14 days of culture, targeted clones were confirmed by PCR using primers REV3-KO GT-Fw and 5′-loxP for knockout and REV3-CD GT-Rv and 3′-loxP for the catalytically dead mutant (Table S1). The PCR products for the catalytically dead mutant were digested with *Nar*I to confirm introduction of the mutations in the 3′-arm. The elimination of exon 5 in mRNA was confirmed by RT-PCR using primers of REV3 ex1 Fw and ex7 Rv. The presence of the designed mutations in the *REV3L* gene was demonstrated by RT-PCR using primers of REV3 mRNA Fw and Rv followed by DNA sequencing.

### Statistical Analysis

Statistical significance was examined by the Student’s *t*-test. Levels of *P*<0.05 were considered to be significant.

## Results

### CGH Array Analyses of Nalm-6 Genome

**Figure 1:**
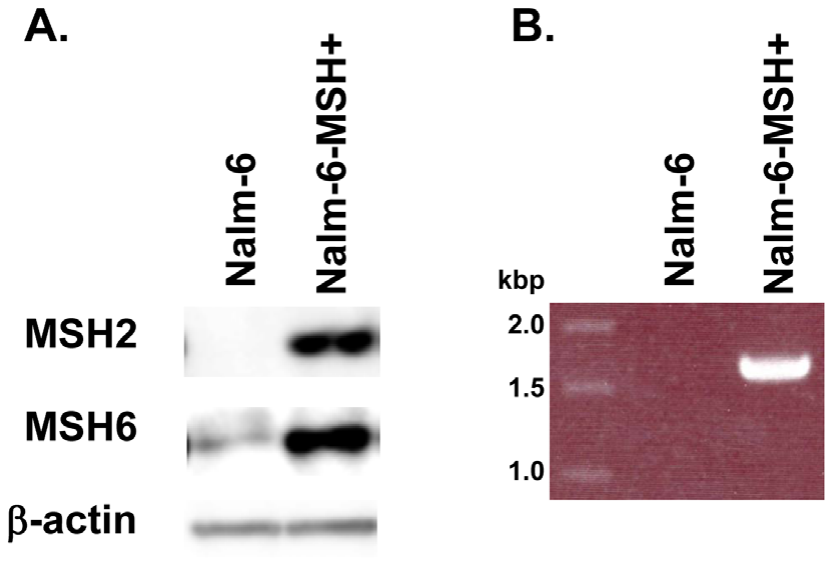
Comparison of Nalm-6 (MSH-) and Nalm-6-MSH+. Western blot analysis of MSH2/MSH6 expression (A) and RT-PCR analysis of MSH2 mRNA from exon 8 to 16 (B).

**Figure 2:**
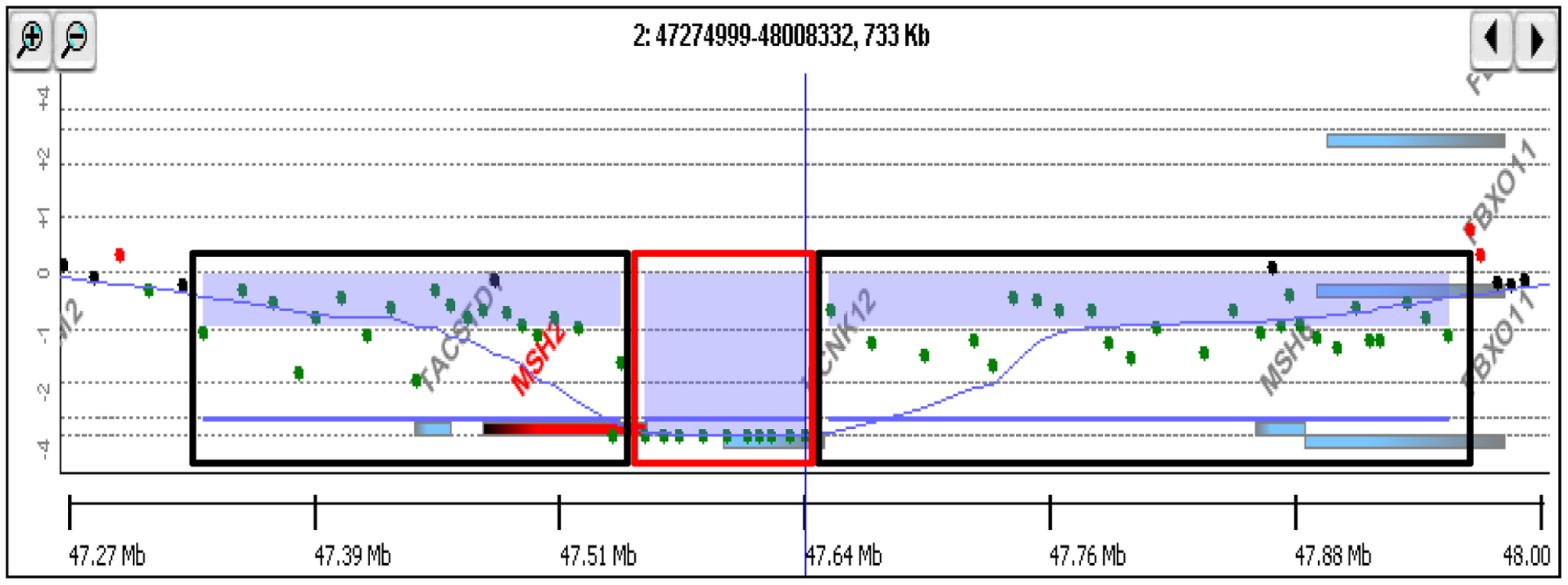
The CGH array analysis of the MSH2 gene in Nalm-6 genome. Part of the chromosome 2 covering the MSH2 gene is presented. The chromosome region where one allele is deleted is boxed with black color and the region where both alleles are missed is boxed with red color. The region covering MSH2 is indicated with a red thick line.

To explore the cause of deficiency in mismatch repair functions in Nalm-6 cells, we first examined the proteins of MSH2 and MSH6 by the Western blotting analysis and confirmed that MSH2 was not expressed and MSH6 was poorly expressed in Nalm-6 (Fig. 1A) [20]. Next, we examined transcripts of the genes by RT-PCR. Although the transcript of the *MSH6* gene was detected (data not shown), no RT-PCR product was detected for *MSH2* gene (Fig. 1B), which were consistent with the previous report [6]. Then, we surveyed mutations in the genomic DNA of Nalm-6 by the CGH array analysis to identify the cause of defect of MSH2 expression. We found that the chromosome 2 had compound heterologous deletions in the *MSH2* gene (Fig. 2). The whole *MSH2* gene was deleted in one allele, while chromosomal region from exon 9 to exon 16 was deleted in another allele. These results indicated that the *MSH2* gene in Nalm-6 completely lost the region between exon 9 and exon 16.

### Restoration of *MSH2* Expression in Nalm-6

**Figure 3:**
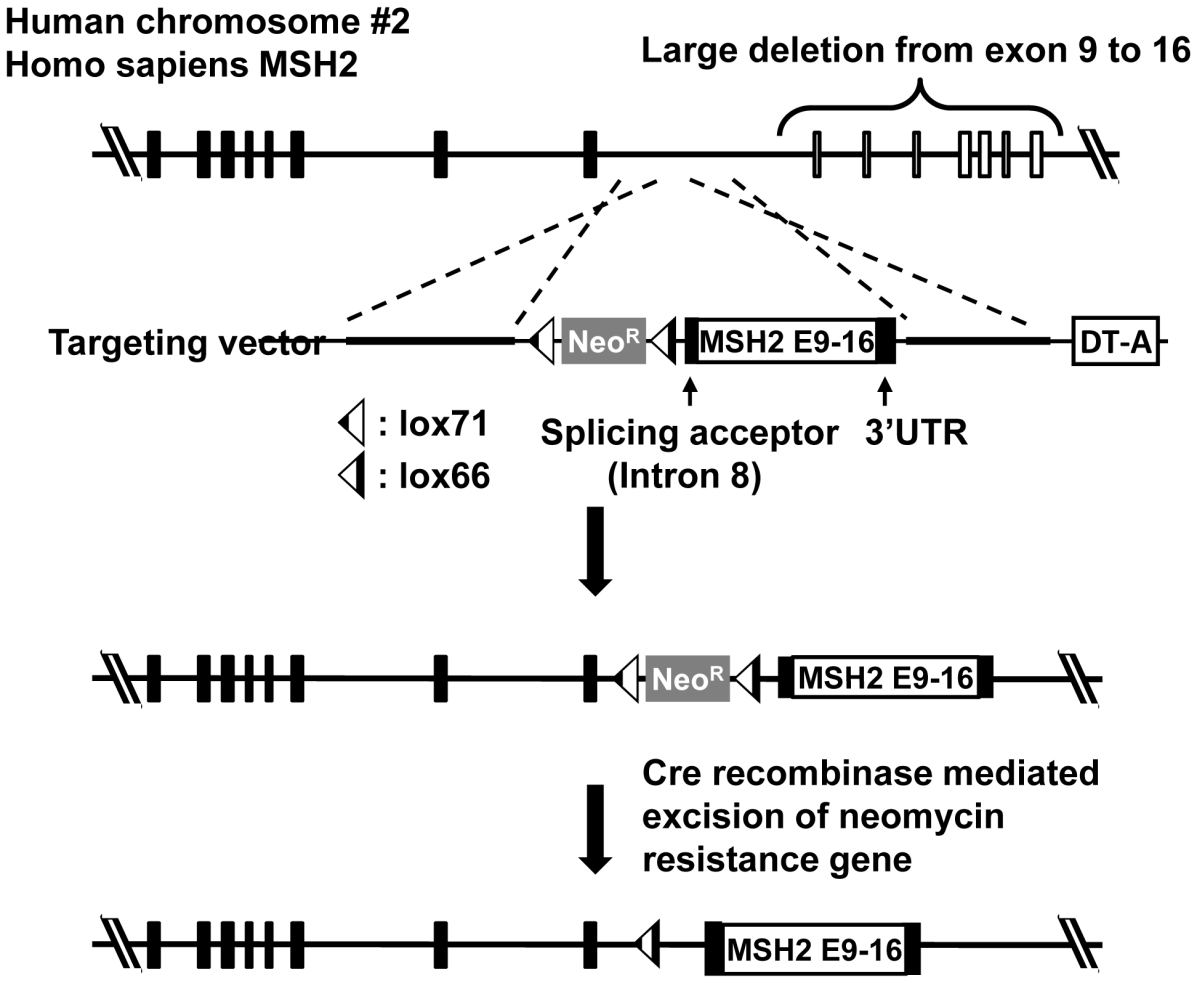
Strategy for re-expression of MSH2 from the endogenous promoter by gene targeting. The MSH2 gene is located on chromosome 2 and the indicated allele has the region from exon 1 to exon 8 of MSH2. Region from exon 9 to 16 is deleted in both chromosomes. The synthetic exon from exon 9 to exon 16 was introduced into downstream of exon 8 by targeting. After Cre recombination, Neo^R^ is removed, and lox71 and lox66 creates a defective lox sequence, which is no longer a target of Cre recombinase. DT-A stands for diphtheria toxin-A.

**Figure 4:**
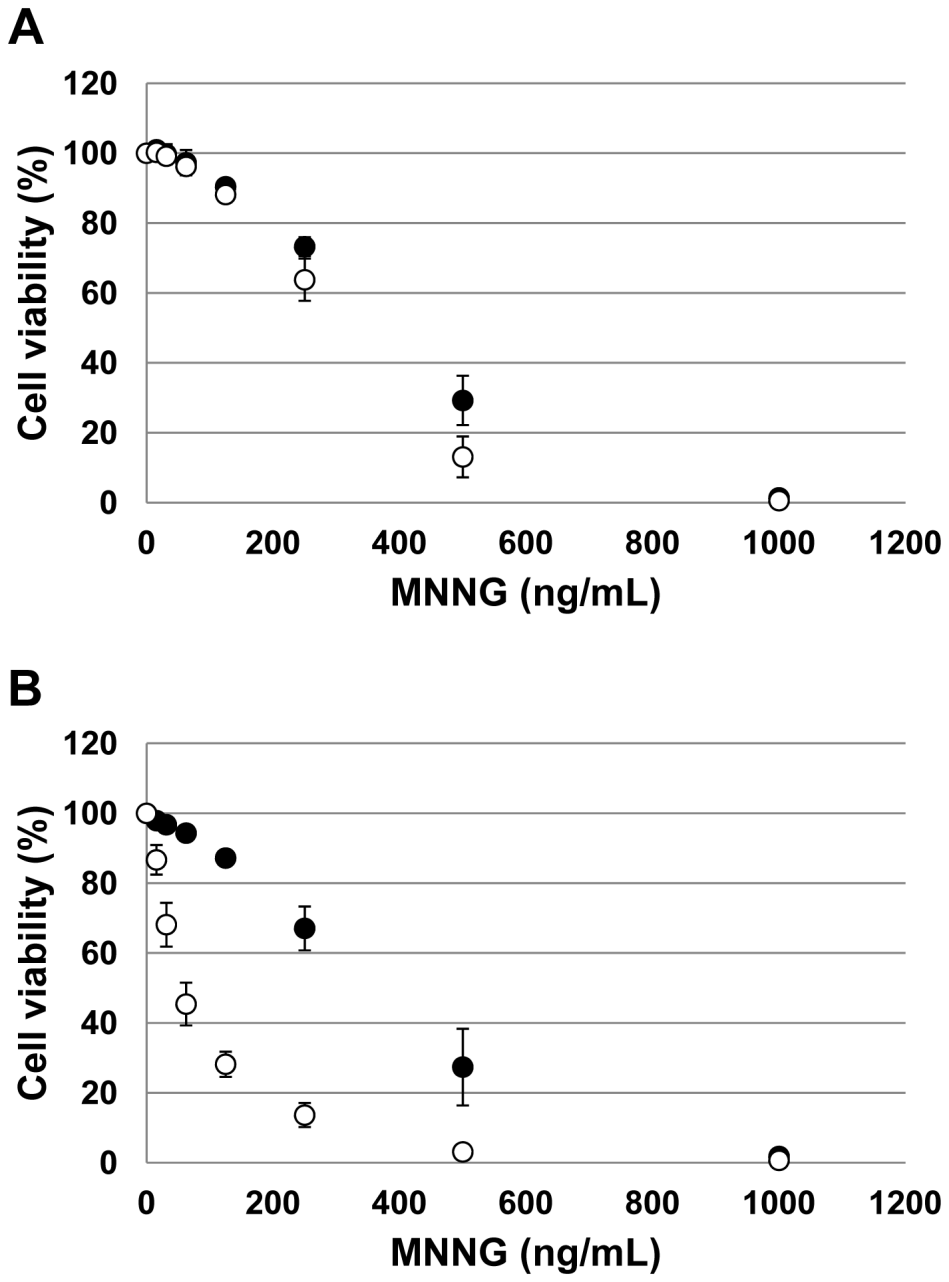
Cytotoxicity of Nalm-6 and Nalm-6-MSH+ cells treated with MNNG. The cytotoxicity in the absence (A) or the presence (B) of O^6^-benzylguanine. Closed and open circles indicate the results of original Nalm-6 and Nalm-6-MSH+, respectively.

To restore MSH2 expression from the endogenous promoter, we introduced the synthetic cDNA sequence corresponding to exon 9 to exon 16 at downstream of exon 8 (Fig. 3). The targeting vector has homology arm for the middle part of intron 8, the neomycin-resistance gene and the cassette containing splicing acceptor of intron 8, artificial exon combined from exon 9 to exon 16 and 3′-UTR region. The neomycin-resistance gene was flanked by two mutant loxP sequences. After the introduction of DNA region from exon 9 to exon 16, the drug resistance gene was cured by transient expression of Cre recombinase. After the gene targeting, the resulting cells transcribed mRNA of the *MSH2* gene and expressed MSH2 protein (Fig. 1 A, B). Furthermore, MSH6 protein was clearly detectable in the targeted cells (Fig. 1A). The growth of the cells expressing MSH2/MSH6, i.e., Nalm-6-MSH+, (21±0.3 h) was slightly slower than the original Nalm-6 cells (19±0.6 h). Spontaneous *HPRT* gene mutation frequency in Nalm-6-MSH+ (3.6±1.8×10^−6^) was substantially lower than that in the original Nalm-6 (96±22×10^−6^). Moreover, the expression of MSH2/MSH6 increased the sensitivity to the cytotoxicity against MNNG in the presence of *O* ^6^-BG (Fig. 4). These results indicated that mismatch repair was functional in the targeted cells.

### Gene Targeting Efficiency

**Figure 5:**
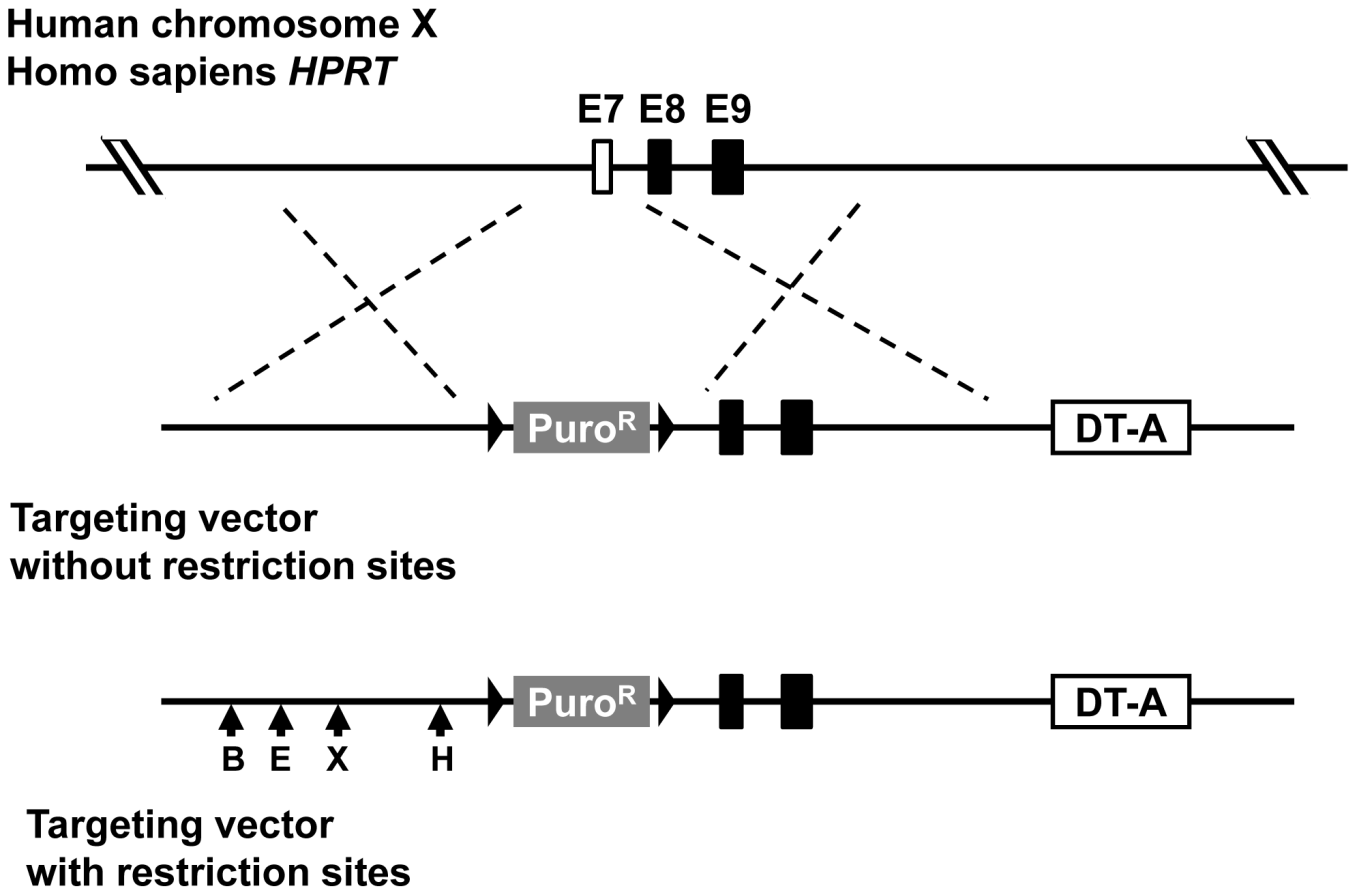
Gene targeting vectors for knockout of the HPRT gene to examine gene targeting efficiencies in Nalm-6 and Nalm-6-MSH+ cells. Part of exon 7 is replaced by the puromycin-resistance gene. One vector (upper) has no mismatch sequence at the 5′-side of the drug-resistance gene and another (lower) has mismatch sequences that create four restriction sites. Closed triangle represents loxP. B; BamHI, E; EcoRI, X; XhoI, H; HindIII.

**Figure 6:**
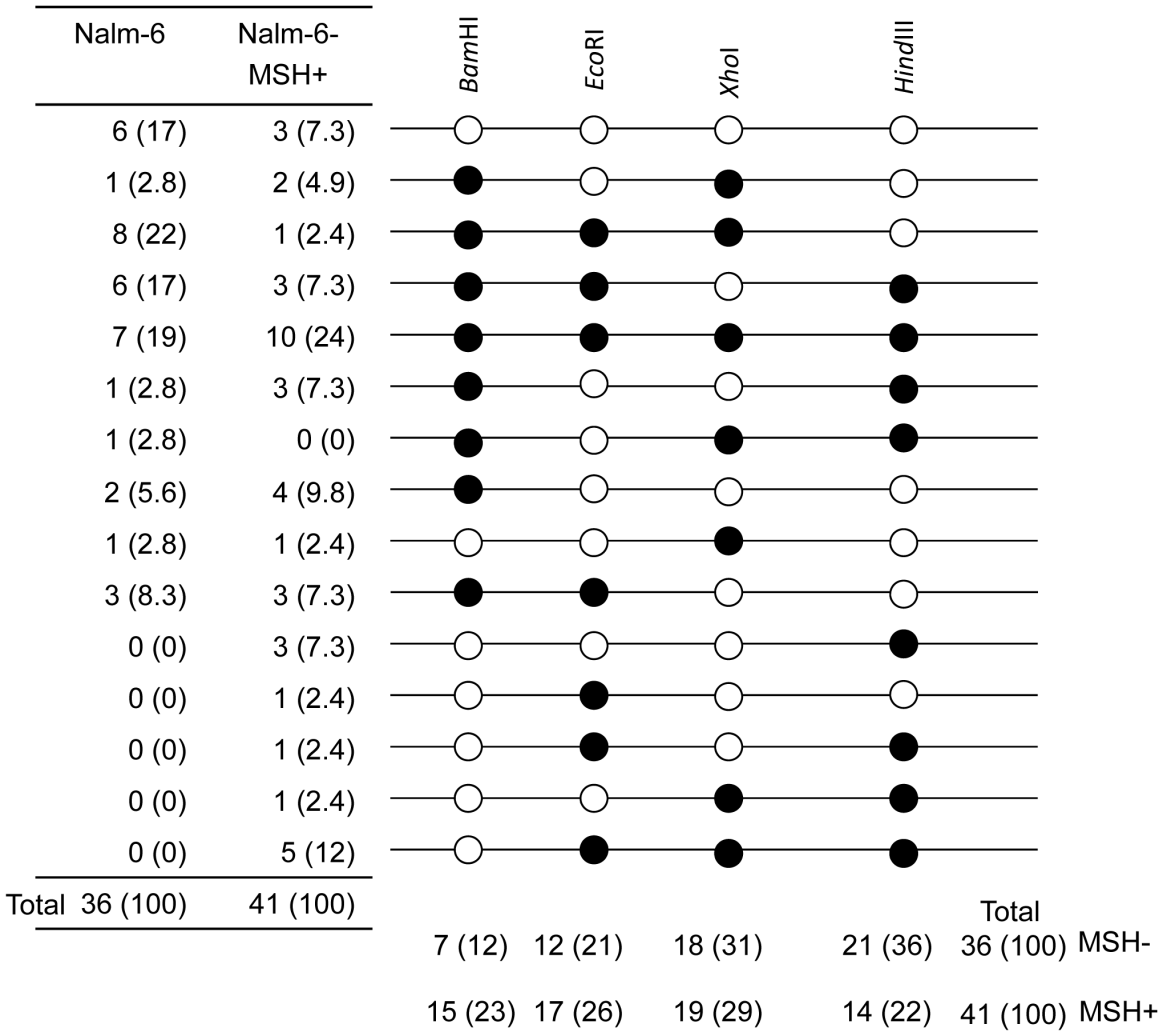
Patterns of restriction marker segregation in 6-thioguanine resistant clones of Nalm-6 and Nalm-6-MSH+ cells. Thirty six and 41, respectively, of 6-thiguanine resistant clones were analyzed in Nalm-6 and Nalm-6-MSH+ cells for the presence or the absence of restriction sites. Open and closed circles represent the presence and the absence of the restriction sites. Figures in parentheses represent percentage of the particular clones.

Mismatch repair is suggested to reduce gene targeting efficiency [27], [28]. We examined the effect of mismatch repair on the efficiency of gene targeting in Nalm-6 with the *HPRT* gene as a model gene (Fig. 5). For knock-in model, four restriction markers were introduced into 5′-arm in the *HPRT* targeting vector. *Hin*dIII, *Xho*I, *Eco*RI and *Bam*HI sites are located 0.56, 1.5, 1.8 and 2.2 kb apart from 3′-end of 5′-arm. First, we examined gene targeting efficiencies with a vector without restriction sites in Nalm-6 and the newly established Nalm-6-MSH+. Random integration frequencies (RIFs), gene targeting frequencies (GTFs) and the gene targeting efficiencies (GTFs/RIFs) in Nalm-6-MSH+ were similar to those in the original Nalm-6 (Table 1). Next, we examined the efficiencies with the vector having four restriction sites in two cell lines. Even though the targeting vector had mismatch sequences, the gene targeting efficiencies were similar in both cell lines (Table 2). The efficiencies were not affected by the presence of mismatch sequences (Table 1 and 2). We also examined whether the restored mismatch repair functions might affect the efficiencies of introduction of small sequence changes, i.e., introduction of restriction sites of *Bam*HI, *Eco*RI, *Xho*I and *Hin*dIII into the chromosome (Fig. 6). About 20% of the targeted cells, which are resistant to puromycin and 6-thioguanine, possessed no new restriction sites in both MSH- cells (the original Nalm-6) and MSH+ cells (19% = 7/36 in MSH- versus 24% = 10/41 in MSH+). Remaining 80% of the targeted cells had one, two, three or four restriction sites. In MSH+ cells, each restriction site was introduced into the chromosome evenly (*Bam*HI, 23% = 15/41; *Eco*RI, 26% = 17/41; *Xho*I, 29% = 19/41, *Hin*dIII, 22% = 14/41) while percentage of introduction of the restriction site slightly increased from *Bam*HI site to *Hin*dIII site in MSH- cells (*Bam*HI, 12% = 7/36; *Eco*RI, 21% = 12/36; *Xho*I, 31% = 18/36, *Hin*dIII, 36% = 21/36). However, the difference between two patterns was marginal and thus we concluded that the restored mismatch repair functions have no substantial effects on repair of the mismatch sequences during gene targeting.

**Table 1:** Gene targeting efficiency at HPRT loci using targeting vectors without mismatch sequences.

		RIFs (×10^−5^)	GTFs (×10^−6^)	GTFs/RIFs (%)
Nalm-6	exp.1	9.4	4.5	4.7
	exp.2	14.7	6.6	4.5
	exp.3	11.4	24.6	21.6
	avg. ± S.D.	11.8±2.7	11.9±11	10.3±9.8
Nalm-6-MSH+	exp.1	4.0	5.3	13.2
	exp.2	12.4	14.1	11.3
	exp.3	6.8	13.5	19.8
	avg. ± S.D.	7.8±4.3	11.0±4.9	14.8±4.4

**Table 2:** Gene targeting efficiency at *HPRT* loci using targeting vectors without mismatch sequences with mismatch sequences.

		RIFs (×10^−5^)	GTFs (×10^−6^)	GTFs/RIFs (%)
Nalm-6	exp.1	13.9	6.7	4.8
	exp.2	13.6	5.8	4.2
	exp.3	4.9	12.9	26.5
	avg. ± S.D.	10.8±5.1	8.5±3.9	11.8±12.7
Nalm-6-MSH+	exp.1	9.7	9.0	9.2
	exp.2	14.1	11.5	8.1
	exp.3	10.6	17.6	16.7
	avg. ± S.D.	11.4±2.3	12.7±4.4	11.3±4.6

### Knockout and Knock-in of the *REV3L* Gene in Nalm-6-MSH+ Cells

**Figure 7:**
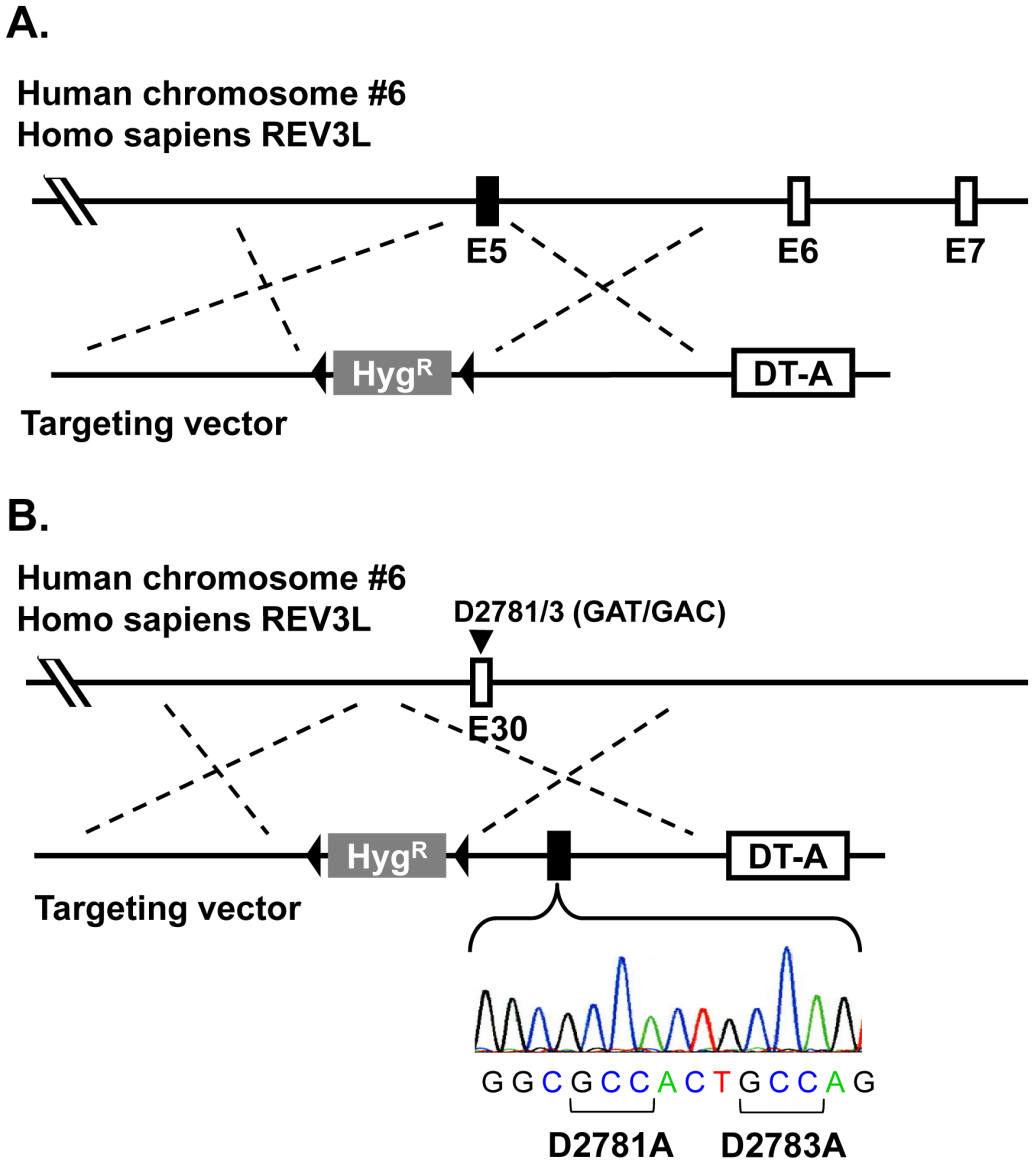
Gene targeting vectors for knockout and knock-in of the REV3L gene. The exon 5 is replaced with the hygromycin-resistance gene for knockout (A). The mutations that direct substitution of amino acids in the catalytic site of REV3L, i.e., D2781A and D2783A, were introduced into exon 30 for knock-in (B).

**Figure 8:**
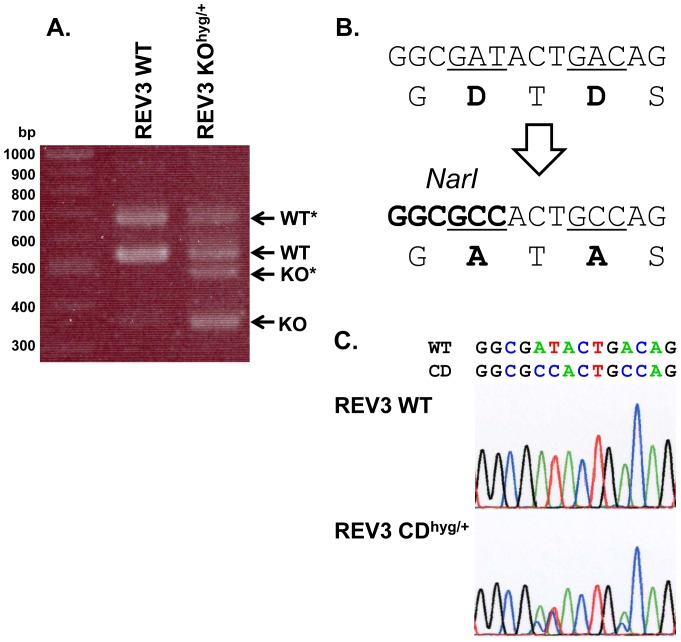
Comparison of the wild-type and a REV3L knockout clone of Nalm-6-MSH+. RT-PCR analysis of mRNA (A). *Splicing variant, which has 128 bp-insertion at downstream of the first exon (35). The design of catalytically dead mutant (B) and cDNA sequence of REV3L knock-in clone (C).

To further demonstrate that Nalm-6-MSH+ cells are useful for gene targeting, we established heterozygous *REV3L* knockout or knock-in cell lines (Table 3). The knockout cell line was generated by replacement of exon 5 of *REV3L* with the hygromycin-resistance gene (Fig. 7A). The knock-in mutation was introduced into exon 30 that results in substitution of amino acids in the catalytic site of DNA polymerase ζ, i.e., D2781A/D2783A (Fig. 7B). Screening 23 hygromycin-resistant clones for *REV3L*-knockout cells resulted in 7 targeted clones, where the exon 5 was replaced with the drug-resistance gene. Therefore, the targeting efficiency was about 30% ( = 7/23) in Nalm-6-MSH+ cells. This value was similar to that in the original Nalm-6-MSH- cells, i.e., 25% = 9 targeted clones/36 hygromycin-resistant clones. Similarly, we obtained 5 targeted clones out of 24 hygromycin-resistant clones for *REV3L*-knock-in cells in Nalm-6-MSH+ cells. Thus, the targeting efficiency was 21%. Two out of five targeted clones had *Nar*I restriction site, which was tracer for alteration of chromosome sequence (Fig. 8 B). This efficiency was similar to that in the original Nalm-6 cells, where 18 positive clones were obtained out of 68 hygromycin-resistant clones. The targeting efficiency was 26% ( = 18/68) in the original Nalm-6 cells. Nine out 18 targeted clones had *Nar*I-sensitive sites. Transcription of knockout and catalytically dead form of REV3L was analyzed by RT-PCR and DNA sequencing (Fig. 8A, C). The short cDNA was detected in heterogeneous knockout clone (Fig. 8A). This result shows that the knockout clone transcribed short mRNA without exon 5. The cDNA sequence of the knock-in clone was a mosaic sequence of the wild-type and the catalytically dead mutant, indicating that the knock-in allele was transcribed. (Fig. 7C). These results clearly indicate that Nalm-6-MSH+ cells can be employed to efficiently disrupt or alter genome sequences in human cells.

**Table 3:** Gene targeting efficiency at REV3 loci using targeting vectors for the knockout mutation (knockout) and the catalytically dead mutation (knock-in).

	Hygromycinresistant colonies	PCR positive colonies	Targeting efficiency (%)	*Nar*I positive colonies	Mutation introducing efficiency (%)
Knockout					
Nalm-6	36	9	25	–	–
Nalm-6 MSH+	23	7	30	–	–
Knock-in					
Nalm-6	68	18	26	9	13
Nalm-6 MSH+	24	5	21	2	8.3

## Discussion

Nalm-6 cells are useful for reverse genetics of human genes because of the high efficiency for gene targeting [6]. However, the cells are defective in MSH2 expression, and thus the spontaneous mutation frequency is very high and they are resistant to cytotoxicity of DNA damaging agents [12]. To suppress the genetic instability and normalize the cellular responses to genotoxic agents, we restored MSH2 expression in Nalm-6 cells. The CGH array analysis clearly indicated that regions covering the whole *MSH2* gene were deleted in one allele while the region covering exon 9 to exon 16 was deleted in another allele (Fig. 2). Introduction of synthetic exons 9 to 16 along with the splicing acceptor site and 3′-UTR at the downstream of exon 8 successfully restored the expression of MSH2 (Fig. 1 and 3). The expression of MSH2 stabilized MSH6, which is unstable in the absence of MSH2, and recovered mismatch repair functions. The spontaneous *HPRT* mutation frequency was reduced more than 25-fold by the expression of MSH2/MSH6. The cells expressing MSH2/MSH6, which we call Nalm-6-MSH+, was more sensitive to the killing effects of MNNG than the original Nalm-6 cells in the presence of *O* ^6^-BG (Fig. 4). These results suggest that the expressed MSH2 acts as a component of mismatch repair and functions as a sensor of DNA damage. The established Nalm-6-MSH+ is more appropriate to examine mutagenicity of genotoxic agents than the original Nalm-6 cells.

Mismatch repair is in general regarded as an inhibitory factor for homologous recombination, which plays a key role in gene targeting (27, 28). The repair enzymes recognize and correct mismatch bases in heterodulplex DNA generated during recombination. It is expected that targeting efficiency with vectors having divergent DNA sequences from the chromosome might be lowered by the presence of mismatch repair systems. In fact, HCT116 human cells, which are sometimes used for gene targeting, are deficient in MLH1, another component of mismatch repair [29]. Thus, we were anxious initially that the recovery of MSH2 expression might reduce targeting efficiency of Nalm-6 cells. As a matter of fact, however, the expression of MSH2 did not suppress gene targeting efficiency with *HPRT* in the cells (Table 1). In addition, MSH2 did not reduce the efficiency with vectors having mismatch sequences in 5′-arm, which is basically homologous to the chromosome (Table 2). These results suggest that the established Nalm-6-MSH+, can be employed in gene targeting with efficiency as much as the original Nalm-6. The reason(s) why mismatch repair did not reduce the targeting efficiency is unclear. However, it is suggested that mismatches in the homology regions of targeting vectors are not seriously deleterious when the percentage of mismatches against the whole homology regions is less than 1% [30]. In the experiments, the percentage of mismatches in the restriction sites against 5′-homology arm of *HPRT* was 0.5% (11 bps/2,398 bps).

REV3L is the gene encoding the catalytic subunit of DNA polymerase ζ, which is a specialized DNA polymerase involved in DNA synthesis across DNA lesions [31]. We chose this gene as an actual target for gene replacement in Nalm-6-MSH+ because the polymerase is expected to be involved in TLS of a variety of genotoxic agents [32]. In addition, the polymerase interacts with multiple other proteins, e.g., REV7/MAD2L2, MAD2 [33], [34] and is twice the size of the yeast homolog mainly because of one exon encoding about 1400 amino acids [35], [36]. Therefore, we speculate that REV3L might have structural roles in defense mechanisms against DNA damaging agents by interacting with other proteins. To distinguish the catalytic and structural roles of DNA polymerase ζ in TLS and other defense mechanisms, we wanted to establish human cells where either DNA polymerase ζ is not expressed (knockout cells) or catalytically-inactive DNA polymerase ζ is expressed (knock-in cells). As the initial approach, we replaced one allele of Nalm-6-MSH+ with targeting vectors for gene knockout and knock-in. For comparison, we also established the same mutants with the original Nalm-6, which is MSH-. As results, both Nalm-6 cell lines exhibited similar high targeting efficiencies for gene knockout and knock-in, i.e., 20 to 25%. These results suggest that Nalm-6-MSH+ cells can be utilized for gene targeting including introduction of small numbers of base substitutions (knock-in) of human genes.

**Figure 9:**
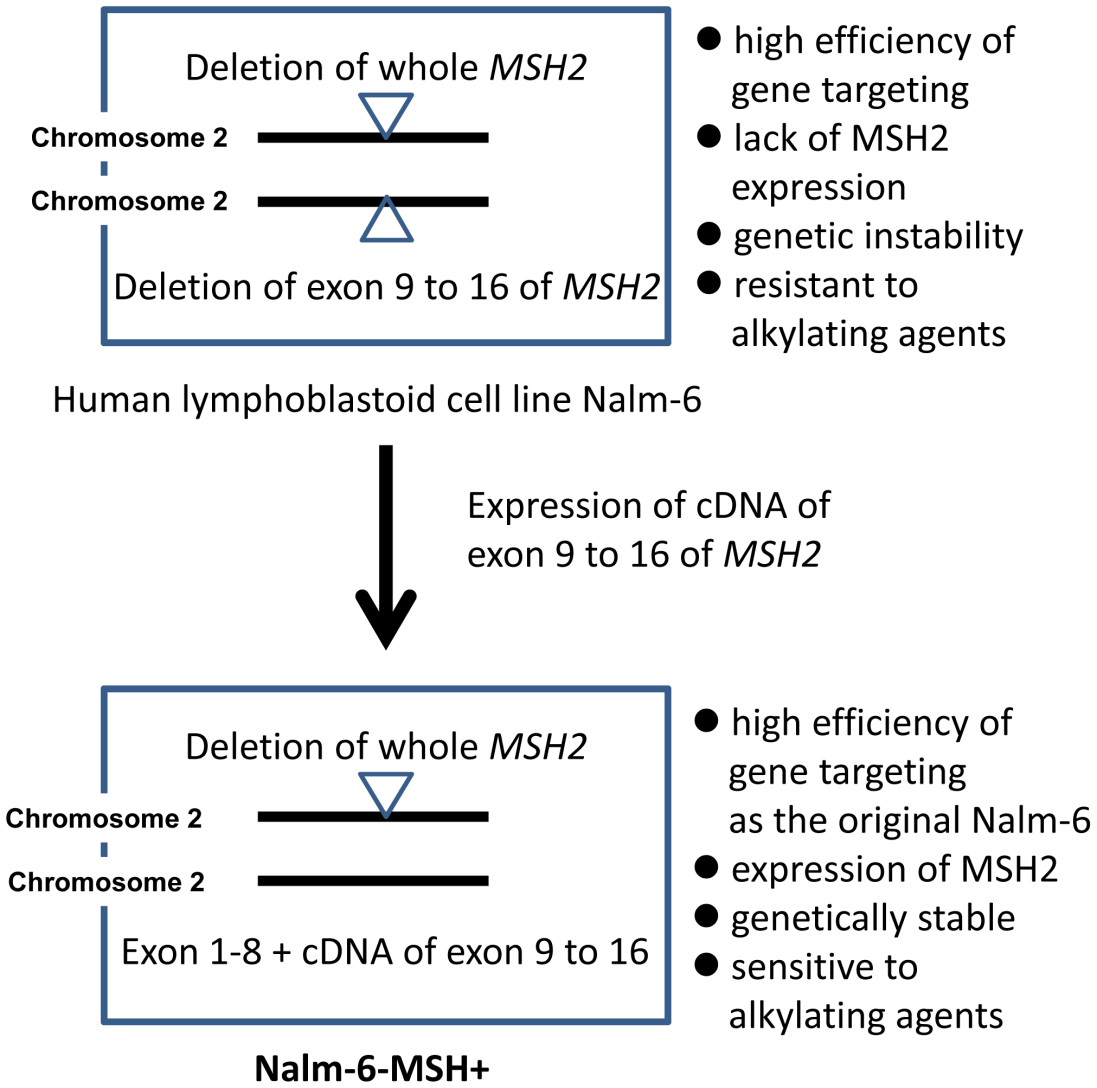
Establishment of Nalm-6-MSH+. The MSH2-expressing cell lime, i.e., Nalm-6-MSH+, has been established by introduction of cDNA of exon 9 to 16 of the MSH2 gene into the original Nalm-6 cells. The resulting cell line exhibits high efficiency of gene targeting as the original Nalm-6 and is genetically stable. It is also resistant to killing effects of alkylating agents.

In summary, we have restored MSH expression in Nalm-6 cell and demonstrated that the mismatch repair functions did not affect high gene targeting efficiencies of the cell line (Fig. 9). The established Nalm-6-MSH+ cells are appropriate for functional analyses of human genes in particular involved in mutagenesis, DNA repair and DNA damage responses. In addition, we demonstrated that not only gene knockout cells but also knock-in mutant cells could be generated by alteration of genome sequences with the cell line. We expect that knock-in strategy will be powerful new tools for studying how gene mutations and variants contribute to susceptibility to diseases and affect responses to therapeutic agents in human cells. The establishment of knock-in mutant cells by amino acid substitutions of target genes enables to analyze precise roles of amino acid sequences in the activity and protein-protein interactions, and effects of SNPs found in cancer cells.

## Supporting Information

Table S1 **A list of PCR primers.**
(DOC)

Method S1 **Construction of pENTR mloxP-Hyg vector.**
(DOC)
