# Supplementary material for: Restoration of Mismatch Repair Functions in Human Cell Line Nalm-6, Which Has High Efficiency for Gene Targeting
Source: PLoS One. 2013 Apr 15;8(4):e61189. doi: 10.1371/journal.pone.0061189 (PMC3626652; doi:10.1371/journal.pone.0061189)
Supplement: Table S1 — A list of PCR primers. (DOC) [file pone.0061189.s001.doc]

| Table S1 | | |
| --- | --- | --- |
| MSH2-E8 Fw | 5’-gtgactcctcttactgatct | Forward primer for amplification of region between exon8 and exon16 |
| MSH2-E16 XhoI-Rv | 5’-gg ctcgag agcccatgggcactgacagt | Reverse primer for amplification of region between exon9 and exon16 with XhoI |
| MSH2-I8 BamHI-Fw | 5’-cc ggatcc aggtagatccttggtttgggcaaca | Forward primer for amplification of region between intron8 and exon9 with BamHI |
| MSH2-E9 Rv | 5’-tcttaccaagatctctggctgcact | Reverse primer for amplification of region between intron8 and exon9 |
| MSH2-E16 Fw | 5’-caggagttcctgtccaaggtgaaac | Forward primer for amplification of region between exon16 and 3’UTR |
| MSH2-3’UTR XhoI-Rv | 5’- gg ctcgag ataccccaccagcccgccgcattta | Reverse primer for amplification of region between exon16 and 3’UTR with XhoI |
| MSH2-5’-arm Fw | 5’-ggggacaactttgtatagaaaagttg atgtgcttgaggagccagagga | Forward primer with attB4 sequence for 5’ arm of MSH2 |
| MSH2-5’-arm Rv | 5’-ggggactgcttttttgtacaaacttg acagttatgcccaatattcattt | Reverse primer with attB1 sequence for 5’ arm of MSH2 |
| MSH2-3’-arm Fw | 5’-ggggacagctttcttgtacaaagtgg cctggtgaaagacaatctcataa | Forward primer with attB2 sequence for 3’ arm of MSH2 |
| MSH2-3’-arm Rv | 5’-ggggacaactttgtataataaagttg tgcctgggctctgggtcccttca | Reverse primer with attB3 sequence for 3’ arm of MSH2 |
| MSH2 GT-Fw | 5’-gcctgtgatcactagtggagaatgtagc | Primer for screening for MSH2 targeting event |
| MSH2-E16 Rv | 5’-agcccatgggcactgacagt | Forward primer for amplification of region between exon8 and exon16 |
| HPRT KO-5’arm Fw | 5’-ggggacaactttgtatagaaaagttg ggttcatctaggttggggcatgta | Forward primer with attB4 sequence for 5’ arm of HPRT |
| HPRT KO-5’arm Rv | 5’-ggggactgcttttttgtacaaacttg catccgtgctgagtgtaccatggt | Reverse primer with attB1 sequence for 5’ arm of HPRT |
| HPRT KO-3’arm Fw | 5’-ggggacagctttcttgtacaaagtgg gctggtgaaaaggaccccacga | Forward primer with attB2 sequence for 3’ arm of HPRT |
| HPRT KO-3’arm Rv | 5’-ggggacaactttgtataataaagttg acctcacgtggagcagataagcaa | Reverse primer with attB3 sequence for 3’ arm of HPRT |
| HPRT-KO GT-Fw | 5’-ctgcaaagcgctgtttcactgttggt | Primer for screening for HPRT targeting event |
| HPRT 5’arm BamHI | 5’-cattttgaggatccgcccgactatt | Primer for introducing BamHI site in 5’ arm |
| HPRT 5’arm EcoRI | 5’-aggcatatgaattcctgagttctct | Primer for introducing EcoRI site in 5’ arm |
| HPRT 5’arm XhoI | 5’-gcctgtgtctcgagtccagcaattc | Primer for introducing XhoI site in 5’ arm |
| HPRT 5’-arm HindIII | 5’-aggagatcaagcttatcctggctaa | Primer for introducing HindIII site in 5’ arm |

| REV3 KO-5’arm Fw | 5’- ggggacaactttgtatagaaaagttg gtgactttgggaacgttaacctcca | Forward primer with attB4 sequence for 5’ arm of REV3 |
| --- | --- | --- |
| REV3 KO-5’arm Rv | 5’- ggggactgcttttttgtacaaacttg ggcaacacagcttatctaagggaag | Reverse primer with attB1 sequence for 5’ arm of REV3 |
| REV3 KO-3’arm Fw | 5’- ggggacagctttcttgtacaaagtgg acgatggttatggacagcagccaga | Forward primer with attB2 sequence for 3’ arm of REV3 |
| REV3 KO-3’arm Rv | 5’- ggggacaactttgtataataaagttg tgatctggaggattggggaagggta | Reverse primer with attB3 sequence for 3’ arm of REV3 |
| REV3 CD-5’arm Fw | 5’-ggggacaactttgtatagaaaagttg ctgtggtctcctgctttccttcag | Forward primer with attB4 sequence for 5’ arm of REV3 |
| REV3 CD-5’arm Rv | 5’-ggggactgcttttttgtacaaacttg gaagcatgctaatgagattttacag | Reverse primer with attB1 sequence for 5’ arm of REV3 |
| REV3 CD-3’arm Fw | 5’-ggggacagctttcttgtacaaagtgg tcatatttgtcaaggcccagcatc | Forward primer with attB2 sequence for 3’ arm of REV3 |
| REV3 CD-3’arm Rv | 5’-ggggacaactttgtataataaagttg ccggctggtaatgaaacagctgctc | Reverse primer with attB3 sequence for 3’ arm of REV3 |
| D2781/3A NarI-S | 5’-gtatatggcgccactgccaggtaatggg | Primer for introducing catalytically dead mutation in 3’ arm |
| D2781/3A NarI-AS | 5’-cccattacctggcagtggcgccatatac | Primer for introducing catalytically dead mutation in 3’ arm |
| REV3-KO GT-Fw | 5’-gtgtgaggtagagctagcggagggaa | Primer for screening for REV3 KO targeting event |
| REV3-CD GT-Rv | 5’-cccttccggctctgacattctgtgagtc | Primer for screening for REV3 CD targeting event |
| REV3 mRNA Fw | 5’-ccgtgtccgtggaaatctcc | Forward primer for amplification of REV3 cDNA around Exon 30 |
| REV3 mRNA Rv | 5’-gaaacagcagggcaggaatc | Reverse primer for amplification of REV3 cDNA around Exon 30 |
| REV3 ex1 Fw | 5’-ggatagtgactgcagactact | Forward primer for amplification of REV3 cDNA around Exon 5 |
| REV3 ex7 Rv | 5’-cggaacttgacagcagccaga | Reverse primer for amplification of REV3 cDNA around Exon 5 |
| 5’-loxP primer | 5’-gataataatggtttcttagacgtgcggc | Primer for screening targeting events |
| 3’-loxP primer | 5’-gaagaggttcactagtactggccattgc | Primer for screening targeting events |
| loxP-RE Fw | 5’-ataacccttaattaccgttcgtataatgt | Primer for mLoxP |
| loxP-LE Rv | 5’-cgagggacctataccgttcgtatagcat | Primer for mLoxP |
