# Supplementary material for: Restoration of Mismatch Repair Functions in Human Cell Line Nalm-6, Which Has High Efficiency for Gene Targeting
Source: PLoS One. 2013 Apr 15;8(4):e61189. doi: 10.1371/journal.pone.0061189 (PMC3626652; doi:10.1371/journal.pone.0061189)
Supplement: Method S1 — Construction of pENTR mloxP-Hyg vector. (DOC) [file pone.0061189.s002.doc]

Method S1

Construction of pENTR mloxP-Hyg vector

The hygromycin-resistance gene flanked with two loxP sites were amplified by PCR using pENTR lox-Hyg as template and mutagenic primers loxP-RE Fw and loxP-LE Rv. The DNA fragment was ligated into pBluescript II SK(+) (Agilent Technologies) at the blunt-SpeI and blunt-PstI sites. The resulting plasmid was named pBS mloxP-Hyg. The plasmid DNA was digested with XbaI/EcoRI. The resulting DNA fragment containing the region from the splicing acceptor site of intron8 to the 3’-UTR was subcloned into pENTR loxP-Hyg at SpeI/EcoRI site. The resulting plasmid was named pENTR mloxP-Hyg.
